# Synthesis and Characterization of Oxidized Polysaccharides for In Situ Forming Hydrogels

**DOI:** 10.3390/biom10081185

**Published:** 2020-08-14

**Authors:** Muhammad Muhammad, Christian Willems, Julio Rodríguez-Fernández, Gloria Gallego-Ferrer, Thomas Groth

**Affiliations:** 1 Department Biomedical Materials, Martin Luther University Halle-Wittenberg, Heinrich-Damerow-Strasse 4, D-06120 Halle (Saale), Germany; muhammad.muhammad@student.uni-halle.de (M.M.); christian.willems@pharmazie.uni-halle.de (C.W.); 2 Centre for Biomaterials and Tissue Engineering, Universitat Politècnica de València, Camino de Vera s/n, 46022 Valencia, Spain; jurodfe2@etsii.upv.es (J.R.-F.); ggallego@ter.upv.es (G.G.-F.); 3 Biomedical Research Networking Centre in Bioengineering, Biomaterials and Nanomedicine (CIBER-BBN), 46022 Valencia, Spain; 4 Interdisciplinary Center of Material Science, Martin Luther University Halle-Wittenberg, D-06099 Halle (Saale), Germany

**Keywords:** alginate, hyaluronic acid, oxidation, in situ gelling, hydrogels, fibroblasts, cytotoxicity

## Abstract

Polysaccharides are widely used as building blocks of scaffolds and hydrogels in tissue engineering, which may require their chemical modification to permit crosslinking. The goal of this study was to generate a library of oxidized alginate (oALG) and oxidized hyaluronic acid (oHA) that can be used for in situ gelling hydrogels by covalent reaction between aldehyde groups of the oxidized polysaccharides (oPS) and amino groups of carboxymethyl chitosan (CMC) through imine bond formation. Here, we studied the effect of sodium periodate concentration and reaction time on aldehyde content, molecular weight of derivatives and cytotoxicity of oPS towards 3T3-L1 fibroblasts. It was found that the molecular weights of all oPs decreased with oxidation and that the degree of oxidation was generally higher in oHA than in oALG. Studies showed that only oPs with an oxidation degree above 25% were cytotoxic. Initial studies were also done on the crosslinking of oPs with CMC showing with rheometry that rather soft gels were formed from higher oxidized oPs possessing a moderate cytotoxicity. The results of this study indicate the potential of oALG and oHA for use as in situ gelling hydrogels or inks in bioprinting for application in tissue engineering and controlled release.

## 1. Introduction

In previous decades, biomedical material research focused on the development of biocompatible and mostly bio-inert biomaterials that do not provoke any adverse reactions in the human body and can be considered as biocompatible [1]. However conventional biomaterials do not always promote healing to the desired extent because of lack of biospecific cues controlling the interaction with the surrounding body fluids, cells, and tissues [2]. More recently, research focused on formation of bioactive and biomimetic materials and surface modifications that communicate in a specific manner with cells at the implantation site, which supports regeneration of tissues [3]. Such products can be based on synthetic materials such as derivatives of poly(ethylene glycol) combined with oligopeptides resembling motifs for cell surface receptors [4]. Another option is based on engineered or naturally occurring polymers, such as elastin peptides [5], fibronectin [6], silk proteins [7], and polysaccharides [8,9] as bioactive materials for making surface coatings or 3D scaffolds and hydrogels.

Polysaccharides (Ps) represent the most abundant biomacromolecules on earth and have been shown to exhibit a wide spectrum of biological effects, namely antioxidant, anticancer, antibiotic, anticoagulant, and bioactive effects on mammalian cells [10]. One of its prominent members is alginate (ALG), a polysaccharide consisting of linear chains of (1→4) β-d mannuronic acid units, α-l guluronic acid units and their corresponding sodium salts [11]. It is used as a biomaterial, due to its excellent biocompatibility and low toxicity. Among other applications, it has been used for the formation of hydrogels and scaffolds for tissue engineering and controlled release systems [12,13]. Hyaluronic acid (HA) is another prominent representative of polysaccharides, specifically of the subgroup of glycosaminoglycans. HA consists of repeating dimeric units of β (1→4) d glucuronic acid and β (1→3) *N*-acetyl-d-glucosamine. HA is the only non-sulfated glycosaminoglycan and widely used in cosmetics, and most importantly, in tissue regeneration applications [14]. Due to their different functional groups, such as hydroxyl and carboxyl groups, polysaccharides can be modified chemically to permit their binding to surfaces or conjugation to other molecules [8]. For example, aldehyde groups can be generated by oxidation of polysaccharides with sodium periodate (NaIO_4_), which splits carbon–carbon bonds between vicinal hydroxyl groups generating dialdehydes [15]. These aldehyde groups can be used to make tissue adhesives that conjugate with amines present on tissue surfaces, or can couple with amino groups of other polysaccharides or proteins to prepare hydrogels [16,17]. 

Hydrogels are used for controlled release of drugs and as scaffolds for soft and hard tissue engineering in regenerative medicine [18]. Special benefits of in situ gelling systems are that they can exactly fill the defects in the host tissue and be loaded with drugs and cells [19]. In addition, such type of hydrogel precursors are also useful as inks for 3D bioprinting [20]. Chemical cross-linked synthetic hydrogels are usually hydrolytic and enzymatic resistant, which might be unfavorable for applications in tissue engineering. On the other hand biopolymers like alginate [21], chitosan [22], hyaluronic acid [23], and other polysaccharides that are degradable can be used for generation of hydrogels to bind adhesions proteins and growth factors for application in tissue engineering [16,24].

In our recent work, we described the oxidation of different polysaccharides by Malparate reaction to obtain reactive derivatives for making of bioactive surface coatings [25] and also for cross-linking reactions with chitosan to prepare hydrogels [16]. Indeed, in the previous studies no systematic approach was taken to study the effect of oxidation condition on aldehyde content and molecular weight of products. The current study aims to create a library of oxidized ALG and HA to study the effect of reaction conditions on degree of oxidation and molecular weight of oxidized alginate (oALG) and hyaluronic acid (oHA). Since both oxidation degree and molecular weight have an effect on biocompatibility, toxicity studies with 3T3 mouse fibroblast were carried out. The ability to apply oALG and oHA as components of in situ gelling hydrogels was tested using carboxymethyl chitosan (CMC) so that both polymer chains can be crosslinked by the formation of imine bonds studying rheological and cytotoxicity properties of the formed hydrogels. 

## 2. Experimental Section

### 2.1. Materials

Native alginate sodium salt low viscosity (Mw ≈236 kDa) was received from Thermo Fisher (Kandel) GmbH (Karlsruhe, Germany). Native hyaluronic acid sodium salt (Mw ≈ 1.2 MDa) was provided by Kraeber & Co. GmbH (Ellerbeck, Germany). Carboxymethylchitosan (CMC, Mw ≈321 kDa, 94.2% degree of deacetylation) was obtained from Heppe Medical Chitosan GmbH (Halle, Germany). Sodium periodate (NaIO_4_ ≥ 99.8%) was purchased from Sigma Aldrich (Steinheim, Germany). Spectra/Por^®^ Dialysis Membrane (Mw CO: 3.5 kD), sodium hydroxide (NaOH), hydrochloric acid (HCl), and potassium chloride (KCl) were obtained from Carl Roth GmbH & Co. KG (Karlsruhe, Germany). Glutaraldehyde solution (50%) was received from Applichem GmbH (Darmstadt, Germany). Schiff’s reagent and hydroxylammonium chloride were purchased from Merck KGaA (Darmstadt, Germany). 

### 2.2. Oxidation of Polysaccharides

Native alginate (nALG) and hyaluronic acid (nHA) were oxidized according to a previous protocol with some modifications [16,26]. A total of 1 g of either nALG or nHA were dissolved in 200 mL of Milli-Q water (0.055 μS cm^−1^). Different amounts of NaIO_4_ were added to the solutions in a flask that was wrapped with aluminum foil (see Section 3.1). The reactions were conducted applying increasing reaction times, while stirring the solutions at room temperature (RT). The oxidized polysaccharides (oPs) were purified by dialysis against distilled water for 3 days, then lyophilized for 24 h (ALPHA 1–2 LDplus freeze dryer, Christ, Osterode am Harz, Germany), and stored at 4 °C.

### 2.3. Characterization of Polysaccharides 

#### 2.3.1. Quantification of Aldehyde Groups in oPs 

Two methods were used to determine the aldehyde content and the degree of oxidation (DO) and degree of substitution (DS) of the different oPs, namely Schiff’s test and titration. oPs were then later denominated according to their oxidation degree as oPS_DS x_.

##### Schiff’s Test

By reacting each of the oPs with fuchsin sulfite reagent (Schiff’s reagent) the molar amount of aldehyde groups can be determined as described previously [27,28]. Aqueous solutions of the different oPs were prepared at a concentration of 4 g L^−1^. In total, 2.5 mL of Schiff’s reagent was added to 500 µL of the sample solution and mixed. The absorbance of the colored complex was measured at 550 nm within 40 min using ultraviolet/visible (UV/VIS) spectrophotometer (Specord200, Analytik Jena AG, Jena, Germany) and compared with a calibration curve made with glutardialdehyde. Three replicates per sample were measured.

##### Titration

A mixture of 25 mL Milli-Q water (0.055 μS cm^−1^) and 20 mL of 0.4 mol L^−1^ hydroxylammonium chloride representing a blank was prepared and its pH was measured. This mixture served as the blank. A total of 60 mg of each oPs was dissolved in 25 mL Milli-Q water and the pH of the solution was adjusted to 7.0 using NaOH (0.01 mol L^−1^). A total of 20 mL of 0.4 mol L^−1^ hydroxylammonium chloride was added and the reaction mixture was stirred for a minimum of 3 h at room temperature. Sodium hydroxide (0.01 mol L^−1^) was used to titrate the released HCl until the pH of the blank was reached. Three replicates of each sample were titrated. Quantification of amino group content of CMC was done by potentiometric titration. Details are shown in the Supplementary Materials Figure S1.

#### 2.3.2. Fourier-Transform Infrared Spectroscopy (FTIR)

FTIR spectra of lyophilized samples were recorded by an ALPHA-Platinum FT-IR Spectrometer with Platinum Diamond-ATR QuickSnap Sampling Module (Bruker, Germany). Measurements were performed in transmittance, 24 scans per sample in the range of 4000–400 cm^−1^ with a resolution of 4 cm^−1^.

#### 2.3.3. Molecular Weight Determination

The weight average molecular weight (Mw) of oPs was determined by gel permeation chromatography at 35 °C using a Waters Breeze GPC system. The equipment has a 1525 Binary HPLC pump (from Waters Corporation, Milford, MA, USA) equipped with a 2414 refractive index detector and four Ultrahydrogel columns connected in series (Ultrahydrogel 1000, 500, 250, and 200). A 150 mM NaCl aqueous solution with 0.05% sodium azide at a flow rate of 0.8 mL min^−1^ was used as the eluent phase. The calibration curve was prepared by using monodisperse polyethylene glycol (PEG) standards of known molar mass at peak, supplied by Waters. The molecular weight of the oPs was referred to the standards. For each measurement, 100 µL of sample was injected at a concentration of 2 mg mL^−1^ in the eluent phase. At least three replicates of each sample were measured.

### 2.4. Cytotoxicity Studies of Oxidized Polysaccharides 

#### 2.4.1. Cell Culture 

3T3-L1 mouse fibroblast cells were used to study the cytotoxicity of oPs solutions as suggested by ISO 10993-3 [29]. Cryopreserved 3T3 fibroblasts were thawed and grown in tissue culture flasks (75 cm^2^, Greiner bio-one, Frickenhausen, Germany) in Dulbecco’s Modified Eagle’s Medium (DMEM) with l-Glutamine and 4.5 g L^−1^ Glucose (Lonza, Verviers, Belgium), supplemented with 10% fetal bovine serum (FBS, Biochrom AG, Berlin, Germany) and 1% penicillin-streptomycin-fungizone (PSF, Promocell, Heidelberg, Germany) in a 37 °C humidified atmosphere of 5% CO_2_ and 95% air using a NuAire DH Autoflow incubator (NuAire Corp., Plymouth, MN, USA). Cells were harvested from pre-confluent cultures using 0.25% trypsin/0.02% ethylenediamine tetraacetic acid (EDTA, Biochrom AG, Berlin, Germany) solution for 3 min at 37 °C. Trypsinization was stopped by the addition of DMEM containing 10% FBS. The cells were resuspended in DMEM with L-glutamine, 10% FBA, and 1% PSF after centrifugation at 250× *g* for 5 min. 

#### 2.4.2. Cytotoxicity Assay 

Ps and oPs were dissolved in phosphate buffered saline (PBS, Merck, Berlin, Germany) at a concentration of 5 mg mL^−1^, afterwards 1 g L^−1^ glucose (Stock solution 100 g L^−1^, Lonza, Verviers, Belgium) and 1% insulin-transferrin-selenium A (ITS, Lonza, Verviers, Belgium) were added. 3T3-L1 cells were seeded at a density of 40,000 cells/well in 96-well tissue culture plates in DMEM supplemented with 10% FBS and 1% PSF and cultured for 24 h. Then the plates were washed once with PBS and 200 µL of each of the oPs and Ps solutions or PBS together with 1 g L^−1^ glucose and 1% ITS (control) were added. Cells were incubated as described above for further 24 h. The viability of cells was measured with QBlue assay according to manufacturer’s instructions (BioChain, Newark, NJ, USA). Before measuring, the solutions of Ps and oPs together with the control were carefully aspirated and the cells were washed with PBS once. Then, 150 µL of pre-warmed DMEM without phenol red, with 4.5 g L^−1^ glucose and without L-glutamine (Lonza, Verviers, Belgium), containing 10% QBlue reagent were added into each well and the samples were incubated again at 37 °C for another 2 h. Thereafter, 100 μL of supernatant of each well was transferred to a black 96-well plate and the fluorescence intensity values were read out at an excitation wavelength of 544 nm and an emission wavelength of 590 nm using a plate reader (FLUOstar Optima, BMG LabTech, Offenburg, Germany). A QBlue solution (10% DMEM) without cells represented a blank value. The viability was calculated as a ratio to the control. Measurements were carried out in pentaplicates. 

#### 2.4.3. Studies on Cell Viability and Morphology Using Vital Staining

Ps and oPs were dissolved in PBS at a concentration of 5 mg mL^−1^. Afterwards, 1 g L^−1^ glucose and 1% ITS were added. 3T3-L1 cells were seeded at a density of 25,000 cells/well in 24-well tissue culture plates in DMEM supplemented with 10% FBS and 1% PSF and cultured for 24 h. Then the plates were washed once with PBS and 500 µL of each of the oPs and Ps solutions or PBS together with 1 g L^−1^ glucose and 1% ITS (control) were added. Cells were incubated as described above for further 24 h. In order to qualitatively evaluate cell viability and the morphology of the cells 5(6)-carboxyfluorescein diacetate (CFDA, Abcam, Cambridge, UK) was used. Briefly, a CFDA working solution (dilution 1/25 in PBS of a stock solution of 3.0 mg CFDA in 1 mL acetone) was prepared. Subsequently, the solutions of Ps and oPs together with the control were carefully aspirated and the cells were washed with PBS once. In total, 200 µL of CFDA working solution was then added to each well. Samples were incubated for 5 min at 37 °C. Afterwards, the CFDA solution was aspirated and replaced by DMEM without phenol red. Viable cells were visualized with a fluorescence microscope (Axiovert 100, 488 nm excitation and 530 nm emission filters, Carl Zeiss MicroImaging GmbH, Jena, Germany) equipped with a CCD camera (Sony, MC-3254, AVT-Horn, Aalen, Germany) and image analyzing software KS300 (Carl Zeiss, Oberkochen, Germany). At least two images using a 10× objective at different locations per well were taken (two wells for each sample, four images in total). 

### 2.5. Mechanical Characterization of Hydrogels by Rheometry

The rheological measurements were carried out on a strain-controlled rheometer (Kinexus lab+, Malvern Panalytical GmbH, Kassel, Germany). Solvent trap geometry of nonporous stainless steel parallel plates of 20 mm diameter was used to avoid sample drying during the experiments. The gap between the plates was fixed to 1 mm. Measurements were performed in shear deformation mode, maintaining the plates at 37 °C. An oscillatory time sweep was recorded to follow the cross-linking process of the hydrogels prepared from oALG_DS 0.25_, oALG_DS 0.49_, oHA_DS 0.02_, and oHA_DS 0.72_, which were dissolved in PBS at 20 mg mL^−1^. CMC was dissolved in PBS at a concentration of 30 mg mL^−1^. A total volume of 340 µL of the aqueous solutions of the hydrogel precursors was arranged on the lower plate, keeping a CMC:oPs volume ratio of 3:1. The required volume of CMC was firstly placed onto the plate, and then the oPs volume was poured with a pipette in the center of the CMC drop. The time evolution of the rheological parameters was recorded for 30 min at a 0.1% strain and 1 Hz frequency to record the gelation kinetics. Two additional experiments were performed after recording hydrogel formation. First, a dynamic strain sweep between 0.01% and 20% at a frequency of 1 Hz was recorded to confirm that 0.1% strain was within the linear region of viscoelasticity of the hydrogels. Second, a frequency sweep between 0.1 and 10 Hz at the fixed strain of 0.1% was registered. Three replicates per sample were measured.

### 2.6. Cytotoxicity Studies of Hydrogels

#### 2.6.1. Cell Culture

Cryopreserved C3H10T1/2 murine cell line (ATCC; LGC Promochem, Molsheim, France) was used to study the biocompatibility of hydrogels. This murine cell line has multi-lineage capacity and was applied to prepare for future studies with these hydrogels in the area of skeletal tissue engineering [30]. The cells were thawed and grown in flasks (75 cm^2^) in DMEM with low glucose (Lonza, Verviers, Belgium), supplemented with 10% heat-inactivated fetal bovine serum and 1% PSF in a 37 °C humidified atmosphere of 5% CO_2_ and 95% air using a NuAire DH Autoflow incubator. Cells were harvested from pre-confluent cultures using 0.25% trypsin/0.02% EDTA solution for 3 min at 37 °C. Trypsinization was stopped by adding DMEM containing 10% heat-inactivated FBS, and the cells were resuspended in Dulbecco’s phosphate buffered Saline (DPBS, Lonza, Verviers, Belgium) after centrifugation at 250× *g* for 5 min. 

#### 2.6.2. Cytotoxicity Assay 

For the cytotoxicity assay of hydrogels, 100 µL of the hydrogel-cell mixture was prepared in 12-well tissue culture plates. For the preparation of the hydrogels, CMC was dissolved in DPBS at a solution of 40 mg/mL^−1^. Then, 56.3 µL of the solution was mixed with 18.7 µL of a cell suspension (100,000 cells) in DPBS. oPS solutions (oALG_DS 0.49_ or oHA_DS 0.72_) with a concentration of 20 mg/mL in DPBS were prepared and 25 µL of the corresponding oPS-solution was mixed with the CMC-cell suspension. As a control a hydrogel based on transglutaminase cross-linked gelatin was used (6% *w*/*v* of G300 gelatin, 10 U of microbial transglutaminase) [31]. After a gelation time of 1 h 1.5 mL of the medium DMEM without phenol red with 1% ITS, 1 g L^−1^ glucose and 1% PSF were added to each well. The viability of cells was measured with a QBlue assay according to manufacturer’s instructions (BioChain, Newark, NJ, USA). Before measuring, the existing medium was carefully aspirated and 1.5 mL of pre-warmed DMEM without phenol red, with 1 g L^−1^ glucose (Lonza, Verviers, Belgium) and containing 10% QBlue reagent was added to each well and the samples were incubated again at 37 °C for another 5 h. Thereafter, 100 μL of supernatant of each well was transferred to a black 96-well plate and the fluorescence intensity values were read out at an excitation wavelength of 544 nm and an emission wavelength of 590 nm using a plate reader (FLUOstar Optima, BMG LabTech, Offenburg, Germany). A QBlue solution (10% DMEM) without cells and hydrogels represented a blank value. 

### 2.7. Statistical Analysis

Data are represented as mean values ± standard deviations. Statistical analysis of all quantitative cell data was performed using Origin 8G (OriginLab Corporation, Northampton, MA, USA) software with one-way analysis of variance (ANOVA), evaluated by post-hoc Tukey’s test. The number of samples is indicated in the respective figure and table captions. Statistical significance was considered for *p* ≤ 0.05 and indicated by asterisks in the figures.

## 3. Results and Discussion

### 3.1. Synthesis and Characterization of Oxidized Polysaccharides 

**Figure 1:**
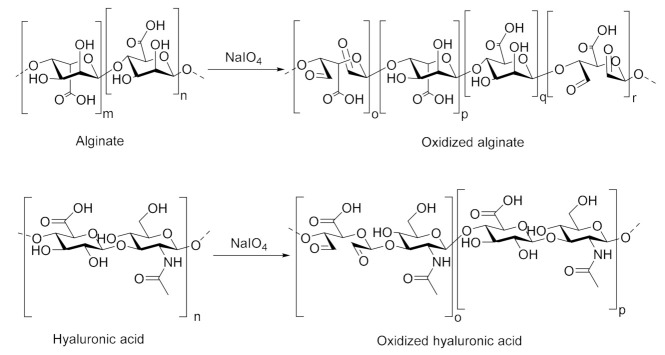
Sodium periodate-mediated oxidation of alginate and hyaluronic acid. The small letters denote the number of repeating units of β-d-mannuronate (*m*), α-l-guluronate (*n*; upper part), and hyaluronic acid (*n*; lower part); the letters *o* and *r* denote the number of repeating units for the units of the polysaccharide chain that were oxidized by NaIO_4_, while the letters *p* and *q* denote the number of repeating units in the polymer chain, that were not oxidized.

Aldehyde groups were introduced to nALG and nHA through oxidation of uronic acid rings by NaIO_4_, by which the vicinal hydroxyl groups on C2 and C3 carbon atoms of β (1→4) linked uronic acid units were oxidized into dialdehydes, thereby opening the sugar rings to form the dialdehyde derivatives oxidized alginate (oALG) and oxidized hyaluronic acid (oHA), respectively (Figure 1). Both of β (1→4) linked mannuronic acid and guluronic acid units can be oxidized in nALG while in nHA oxidation is only possible in glucuronic acid units.

Different amounts of NaIO_4_ and different reaction times were applied to oxidize nALG and nHA in order to obtain oPs with different oxidation degrees (Table 1 and Table 2). In the case of nALG oxidation, shorter reaction times (3 h) were applied when compared to nHA (6 h) because it has a lower Mw than nHA and accordingly a lower tendency of the molecule to form coils in solution due to intramolecular hydrogen bonding; hence, NaIO_4_ can access the vicinal hydroxyl groups in nALG more easily [32].

The aldehyde groups obtained by oxidation in each oPs were quantified to determine the corresponding DO_exp_ and DS_CHO_ (Table 1 and Table 2). DO_exp_ is the percentage of the experimental aldehyde group concentration obtained from oxidation of Ps in relation to its maximum corresponding theoretical concentration. DS_CHO_ represents the amount of the hydroxyl groups, which has been oxidized in one saccharide repeating units of the corresponding polysaccharide. DS_CHO_ has a maximum value of 2 because only two aldehyde groups can be generated in each saccharide repeating unit by the oxidation process. The results obtained from titration were slightly higher in most of the cases in comparison to Schiff’s reagent results. This can be explained by the higher sensitivity of the titration method to quantify the aldehyde groups in oPs. In the case of nALG, the aldehyde content increased when higher concentrations of NaIO_4_ were used. However, extending the reaction time from 3 h to 24 and 72 h to ensure complete cleavage of vicinal hydroxyls had only minimal effect on the aldehyde content (Table 1).

Regarding nHA oxidation (Table 2), the DO_exp_ reached only 1%, when an amount of 0.5 eq NaIO_4_ was added to oxidize the vicinal hydroxyl groups. A possible reason for this observation is that these groups in nHA are hindered by hydrogen bonds and thus are less sensitive to the oxidizing reagent [32]. The aldehyde content in oHA increased remarkably when the reaction time was prolonged from 6 to 24 h. At a reaction time of 24 h, there was almost no difference in the DO_exp_ and DS_CHO_ visible for samples functionalized with 1.0 and 1.2 eq of NaIO_4_. However, a maximum DO_exp_ and DS_CHO_ were obtained for oHA when the reaction time was extended to 72 h and 1.2 eq of NaIO_4_ were used. This could be attributed to the fact that increasing the reaction time allows NaIO_4_ to degrade the nHA molecule resulting in fragments of lower molecular weight and thus facilitating the oxidation process [33]. The reduction in molecular weight was indeed observed during gel permeation chromatography (GPC) studies (Supplementary Materials Tables S1 and S2).

**Figure 2:**
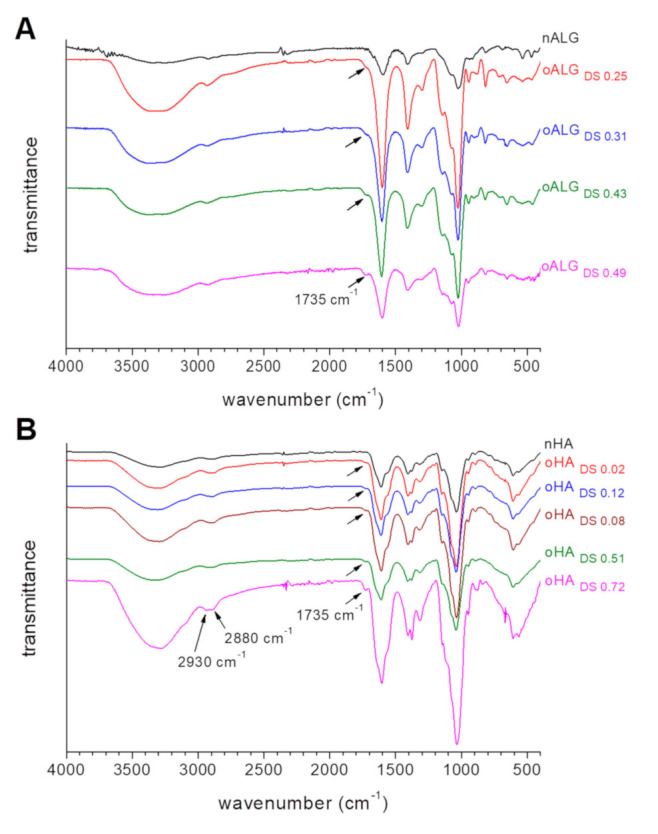
ATR-FTIR spectra of native and oxidized derivatives of (**A**) alginate and (**B**) hyaluronic acid. The curves are vertically shifted for a better comparison.

FTIR spectra of the samples (Figure 2) showed that in addition to the characteristic peaks, there is a signal at 1735 cm^−1^ corresponding to aldehyde groups in oHA and in oALG compared to their native counterparts. 

The FTIR spectrum of native alginate in Figure 2A presents the characteristic carboxylate (‒COO^−^) vibrational modes, antisymmetric stretch at 1596 cm^−1^, and symmetric stretch at 1406 cm^−1^. Bands assigned to the symmetrical C–O–C stretching of the acetal group (1081 and 814 cm^−1^) and the anti-symmetrical C–O–C stretch (1027 cm^−1^) are also observed [34]. The spectra of oxidized alginate (Figure 2A) confirmed the presence of aldehyde groups in oALG by the new band at 1735 cm^−1^, with an intensity that increases with the oxidation degree [35]. 

FTIR spectra of native hyaluronic acid and its oxidized derivatives are represented in Figure 2B. The spectrum of native hyaluronic acid shows the peak at 1040 cm^−1^ attributed to C-O-C vibration, and the peaks at 1640 and 1411 cm^−1^ attributed to the asymmetric bending and symmetric stretching of C=O groups in nHA, respectively [36]. Again, a new band at 1735 cm^−1^ is observed in the oHA confirming the presence of aldehyde groups due to oxidation. The intensity of this peak also increases with the oxidation degree, indicating successful reaction. It is worth noting that the spectrum of the sample with the higher oxidation degree (oHA_DS0.72_) showed two absorption peaks at 2930 to 2880 cm^−1^, which corresponded to the generation of the dialdehyde group [33]. This double peak was not observed for lower oxidation degrees.

**Figure 3:**
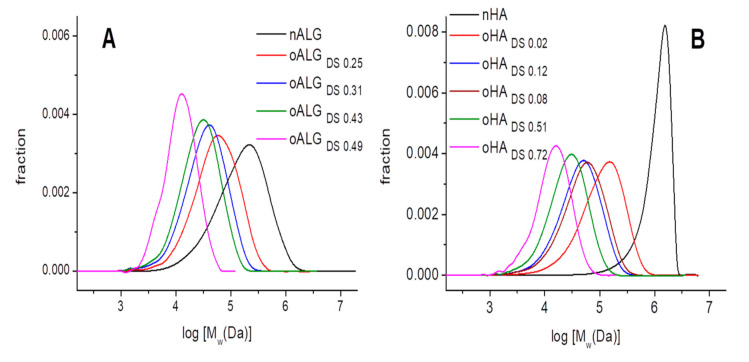
Molecular weight distribution of native and oxidized derivatives of (**A**) alginate and (**B**) hyaluronic acid as determined by gel permeation chromatography (GPC).

The molecular weight distribution curves plotted in Figure 3 show the reduction of the molecular weight with the oxidation degree (mean Mw values and PDIs can be found in Supplementary Materials Tables S1 and S2). nALG had a broad molecular weight distribution curve (Figure 3A) with weight-average molecular weight (Mw) of 236 kDa and a polydispersity index of 2.9. As the oxidation degree increased, the distribution curve moved to lower molecular weights, while becoming less wide (Figure 3A). Consequently, the measured Mw values became lower, up to a mean value of 14 kDa for the higher oxidation degree (oALG_DS 0.49_). The polydispersity index decreased with the oxidation degree (Supplementary Materials Table S1), except for the oALG_DS 0.49_ where two populations of different Mw seemed to appear, one of which had a lower Mw; observed as a shoulder of the main peak in the distribution curve of Figure 3A. 

Concerning hyaluronic acid, nHA had a large Mw, 1224 kDa, and a PDI of 2.1. Oxidation process had a high impact in the reduction of its molecular weight and even at low oxidation degrees (DS 0.02) the distribution curve moves to quite low molecular weight (Mw = 148 kDa). The evolution of the molecular weight distribution curves was similar to what was observed in oALG (Figure 3B), the higher the oxidation degree the lower the Mw while the PDI stays at almost a constant value (Supplementary Materials Table S2).

Dialdehyde alginate acid and hyaluronic acid chains generated by oxidation with sodium periodate had low stability in alkali media and chain scission tended to occur from the glycosidic bond [35]. Moreover, it was found that the oxidation of alginate altered the chain conformation and the glycosidic linkage became more susceptible to hydrolysis [37], which might have contributed to the reduction of the molecular weight of the oxidized derivatives. 

### 3.2. Cytotoxicity of Oxidized Alginate and Oxidized Hyaluronic Acid 

**Figure 4:**
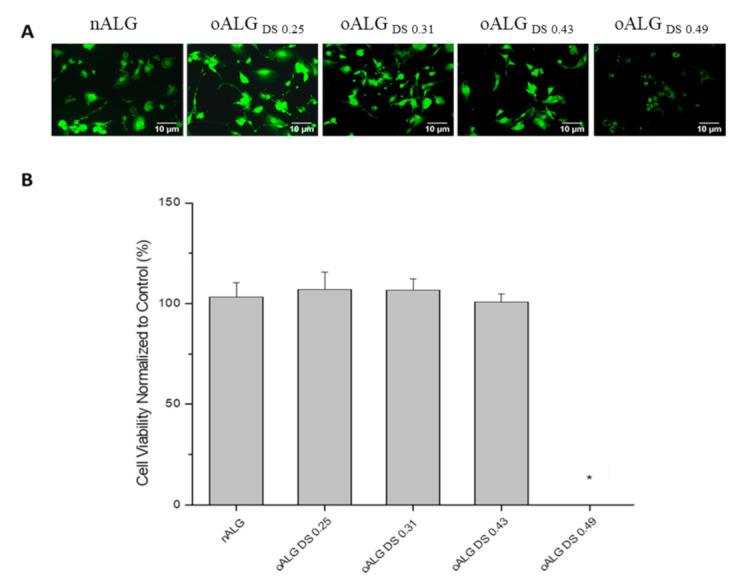
(**A**) Fluorescence micrographs after staining with vital stain 5(6)-carboxyfluorescein diacetate (CFDA) and (**B**) viability studies by Q-blue assay of 3T3-L1 fibroblasts incubated in native and oxidized alginate solutions in phosphate buffered saline (PBS) with a concentration of 5 g L^−1^ for 24 h; scale bar: 10 μm. Data represent mean ± SD values normalized to control, *n* = 10, * *p* ≤ 0.05; compared to native alginate (nALG).

**Figure 5:**
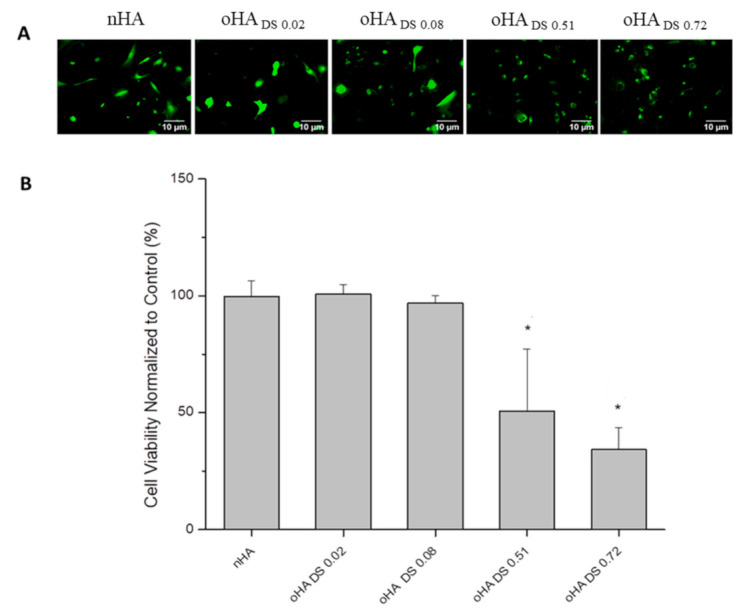
(**A**) Fluorescence micrographs after staining with vital stain CFDA and (**B**) viability studies by Q-blue assay of 3T3-L1 fibroblasts incubated in native and oxidized hyaluronic acid solutions in PBS with a concentration of 5 g L^−1^ for 24 h; scale bar: 10 μm. Data represent mean ± SD values normalized to control, *n* = 10, * *p* ≤ 0.05; compared to native hyaluronic acid (nHA).

Cytotoxicity studies were conducted with the QBlue assay measuring the metabolic activity of cells after incubation with the different Ps and oPs. Regarding alginate, there was no cytotoxicity observed when nALG and oALG with different substitution degrees were incubated with the cells, except for the oALG with the highest degree of substitution (oALG_DS 0.49_), which had a very high toxic effect (Figure 4B). The results obtained from Qblue were confirmed with cell viability staining using CFDA (Figure 4A). The micrographs showed a very low fluorescence intensity of the cells in the case of oALG_DS 0.49_, which indicated that the fibroblasts were greatly damaged. On the other hand, the fluorescence intensities were high and cell spreading was observed when nALG and the other oALG with different DSs were incubated with the cells. It was clear that the oxidation of nALG had no effect on the toxicity of 3T3-L1 fibroblasts until a specific DS. After this critical DS was reached, the cells lost their metabolic activity. Therefore, there was no correlation between the CHO content of ALG and the metabolic activity of 3T3-L1 fibroblasts (Supplementary Materials Figure S2). The strong cytotoxic effect of oALG_DS 0.49_ is probably related to the low molecular weight of the product compared to the other oALG products. We found similar toxic effects of lower molecular weight oxidized cellulose sulfates in a previous study [38]. However, there was a significant relationship between the CHO content of HA and the metabolic activity of 3T3-L1 fibroblasts (Supplementary Materials Figure S3). When nHA and oHA with low DSs (oHA_DS 0.02_ and oHA_DS 0.08_) were incubated with the cells, no cytotoxicity was observed, whereas the metabolic activity of the cells became lower in the case of oHA_DS 0.51_ and oHA_DS 0.72_ (Figure 5B). Similar results were obtained with CFDA viability staining, showing that the fluorescence intensities were relatively low when the cells were incubated with both oHA_DS 0.51_ and oHA_DS 0.72_, indicating cytotoxicity, while the cells were still intact and showing higher fluorescence intensities and spreading when the cells were incubated with nHA, oHA_DS 0.02_, and oHA_DS 0.08_ (Figure 5A). 

The cytotoxic effects from both oALG and oHA can be attributed to the reactive aldehyde groups generated during the oxidation process. These aldehyde groups might have bonded with the primary amine functional groups of proteins, which are found in the cell membrane of the biological system. This binding causes damage to the cell membrane [39]. It is also worth noting that the cytotoxic effect increased when the DS of oPs was higher. The relatively low Mw of the oPs is another factor that might have led to cytotoxicity in an indirect manner. When the Mw of oPs is low, the availability of the reactive aldehyde groups is higher because of the low number of intramolecular hydrogen bonds that can occur and consequently hinder the accessibility of these groups [32]. This might explain why oALG_DS 0.49_ had a high cytotoxic effect while oALG_DS 0.43_ was biocompatible when incubated with cells even though both had relatively close DSs. Furthermore, oHA, exactly similar to nHA, can still bind to the HA cell surface receptors for endocytosis and consequently be absorbed into the cell through the endocytosis mediated by the hyaluronic acid receptor (CD44) [40,41]. Inside the cells, the aldehyde groups of oHA might have bonded with the amine groups of different amino acids, proteins, and other cell components, which could have damaged the cells and caused cytotoxicity. In the case of oALG, cells do not have receptors that recognize ALG and accordingly oALG cannot enter the cells by endocytosis [42]. In addition, it is unlikely for oALG and oHA with different DSs to diffuse across the cell membrane because their Mw range is still considered relatively high (from 10 to 148 kDa) [43]. 

### 3.3. Rheological Properties of oPs/CMC-Hydrogels

**Figure 6:**
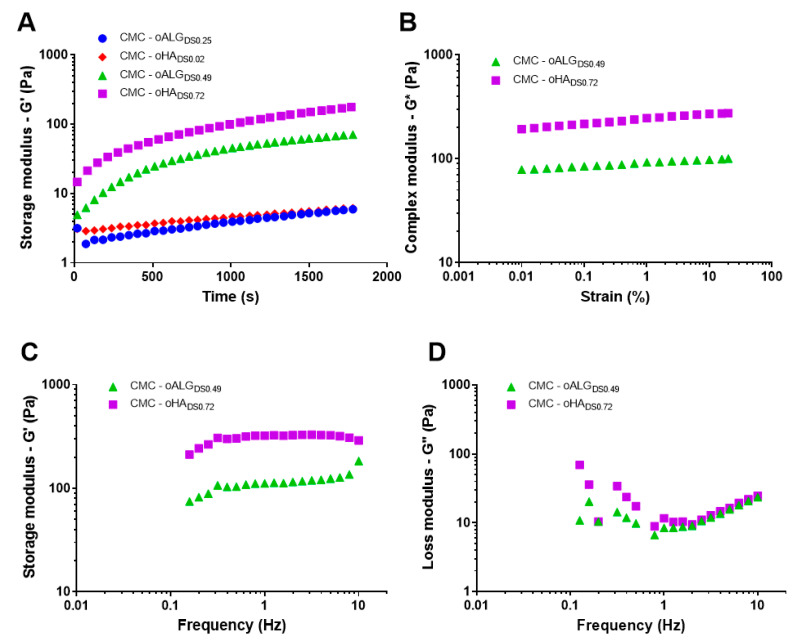
Rheological properties of the hydrogels at 37 °C. Each curve corresponds to the average of three different samples. (**A**) Crosslinking process represented with storage modulus as a function of reaction time at a frequency of 1 Hz and 0.1% strain. (**B**) Dependence of the complex modulus (G*) on the strain amplitude at a frequency of 1 Hz of the already crosslinked hydrogels, measured after the crosslinking process. (**C**,**D**) Evolution of the storage (G’) and loss (G”) moduli, respectively, as a function of the frequency at 0.1% stain of the crosslinked hydrogels.

Selected oPs with different degrees of oxidation were mixed with CMC and their rheological properties were analyzed to determine if the imine formation between CMC and the oPs takes place and a stable hydrogel is formed. Figure 6A shows hydrogels crosslinking dynamics, followed by the evolution of the shear storage modulus (G’) with the reaction time. For low oxidation degree samples (CMC-oALG_DS 0.25_ and CMC-oHA_DS 0.02_), a very slight increase in the shear storage modulus was observed with the reaction time, indicating that hydrogels were not crosslinked after 30 min within the rheometer. On the contrary, successful crosslinking was observed for highly oxidized polysaccharide hydrogels (CMC-oALG_DS 0.49_ and CMC-oHA_DS 0.72_), as the storage modulus sharply increased during the gelation dynamics and almost stabilized during the time of the experiment. By the end of the measurements, a small slope is observed in G′, indicating that the crosslinking reaction is still taking place. The dependence of the slope on reaction time dG’(t)/dt was determined and the stabilization time was calculated as the crossing point of the initial and final slope [44]. The fastest dynamic was observed for CMC-oHA_DS 0.72_ that resulted in a gelation time of 8 min (Table 3), while 16 min were necessary for CMC-oALG_DS 0.49_ hydrogel to stabilize and form a gel (Table 3). The faster gelation is probably due to the higher oxidation degree of oHA_DS 0.72_ compared to oALG_DS 0.49_, which accelerated the reaction with the amine groups of CMC.

Figure 6B shows that the complex modulus of the already crosslinked hydrogels remained independent of the strain amplitude, indicating that the measuring conditions were in the linear viscoelastic range of the hydrogels [45] and that the selected 0.1% strain for the measurements of Figure 6A was valid. Subsequent frequency sweep tests on the already crosslinked hydrogels were then performed at 0.1% strain amplitude. Figure 6C,D depicts, respectively, the dependence of storage (G’) and loss (G”) moduli on the frequency. G’ values were much higher than G” values at least for frequencies higher than 1 Hz, as usually observed in elastic hydrogels [45]. As seen in Figure 6C, the storage modulus G’ remains constant for a wide range of frequencies, as corresponds for the rubbery plateau region. We mechanically characterized the hydrogels by tabulating G’ and G” values at 1 Hz (Table 3). CMC-oHA_DS 0.72_ had a storage modulus of 323 Pa, which was stiffer than CMC-oALG_DS 0.49_ hydrogel that showed a storage modulus value of 111 Pa. This was a consequence of the higher oxidation degree of oHA_DS 0.72_ in comparison to oALG_DS 0.49_ that resulted in a more crosslinked network by the formation of more bonds with the CMC chains. 

### 3.4. Cytotoxicity of Hydrogels Made of Oxidized Alginate and Oxidized Hyaluronic Acid 

**Figure 7:**
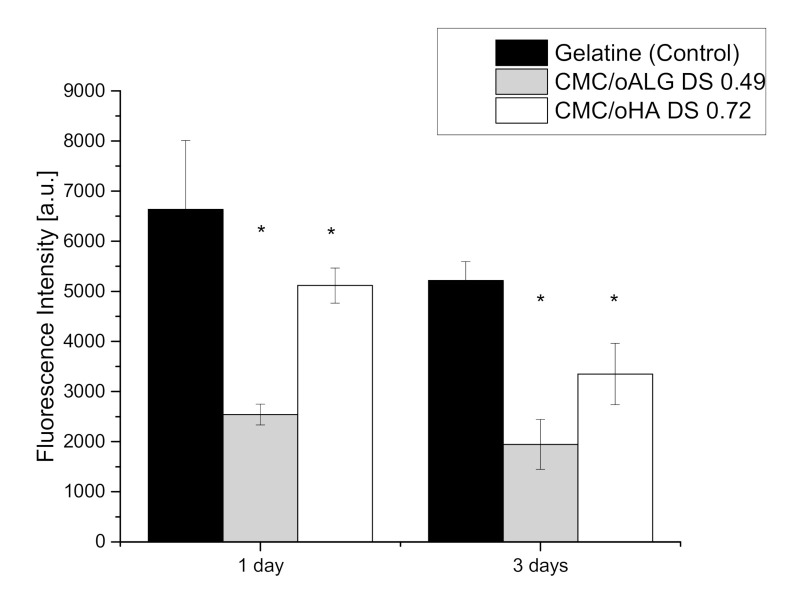
Viability studies by Q-blue assay of C3H10T1/2 mouse stem cells incubated in hydrogels, containing carboxymethyl chitosan (CMC) and oxidized hyaluronic acid or oxidized alginate for 24 and 72 h. Data represent mean ± SD values normalized to control, *n* = 4, * *p* ≤ 0.05.

As with the single components of the hydrogel, the cell viability of hydrogels comprised of CMC and oxidized hyaluronic acid or oxidized alginate was also studied with the QBlue assay measuring the metabolic activity of cells embedded in the hydrogels after incubation for 1 and 3 days. Here, the cell viability of C3H10T1/2 mouse stem cells was investigated because we intend to investigate cell differentiation in these hydrogels in future studies. The hydrogels containing the highest amount of aldehyde groups in its oxidized polysaccharide components were chosen as to guarantee the highest possible gel stability. As a control, a gelatin hydrogel cross-linked enzymatically was applied since it is known for its general biocompatibility and low cytotoxicity [31]. The results are shown in Figure 7.

It is apparent that cells in both oPs hydrogel samples show a lower fluorescence intensity than those in the control sample gelatin hydrogel, corresponding to a lower number of living cells after 1 and 3 days. This suggests that the chemically cross-linked oPs hydrogels have a lower biocompatibility than gelatin hydrogels as it could be also expected from toxicity measurements in Section 3.2. However, the viability of cells in hydrogels seems to be less affected compared to the exposure of highly oxidized oALG and oHA in soluble form indicating that cross-linking with CMC consumes aldehyde groups, providing a protective effect. It is also visible that viability of cells was higher in CMC-HA_DS 0.72_ based hydrogels though oxidation degree of oHA was higher than that of oALG. Another observation is the small decline of viability of cells from day 1 to day 3 in oPs based hydrogels, which we believe is due to lack of adhesive cues provided by the polymeric network towards cellular integrin adhesion receptors. This suggests that the degree of oxidation is not the only determining factor of viability of cells embedded in the hydrogel. 

## 4. Conclusions

In this study, two different polysaccharides, namely alginate and hyaluronic acid were oxidized to obtain oPs with different oxidation degrees. The two varying parameters were the amount of NaIO_4_ that was used and the reaction time. In the case of ALG, oxidation degrees between 12.5% and 24.5% were obtained. The degree of oxidation could mainly be influenced by changing the amount of NaIO_4_, while the influence of the reaction time was found to be minimal. Regarding HA, the degree of oxidation was dependent on both factors. Higher amounts of NaIO_4_ in combination with a prolongation of the reaction time to at least 24 h led to significant degree of oxidation higher than 25%. It was also found that the Mw of oALG and oHA decreased when their respective oxidation degrees were higher. A reduction in Mw from 236 to 16 kDa was found when nALG was oxidized to obtain oALG with the highest oxidation degree (oALG_DS 0.49_). In the case of HA, the oxidation process had a higher impact on the reduction of the molecular weight compared to ALG. The relatively high Mw of nHA (1224 kDa) decreased by a factor of 10 for the lowest degree of oxidation to a factor of 100 for the highest degree. Studies with 3T3-L1 fibroblasts showed that oPs with higher oxidation degrees (oALG_DS 0.49_, oHA_DS 0.51_, and oHA_DS 0.72_) reduce the metabolic activity tremendously. This toxic effect can be attributed to the free aldehyde groups generated during oxidation and the smaller size of products that may lead to membrane permeabilization of cells. Hydrogels could be prepared by crosslinking aldehyde groups from oALG and oHA with amine groups present in CMC. Successful crosslinking was confirmed with rheological studies and the fastest gelation dynamics were recorded when oHA_DS 0.72_ and oALG_DS 0.49_ were used to prepare hydrogels, respectively. Toxicity studies of the hydrogels showed a lower toxicity for cells in oHA based hydrogels, compared to oALG based hydrogels. Hence, this study shows that the oPS members of the generated library are suitable for formation of in situ gelling hydrogels, which also makes them applicable as inks for bioprinting processes including bioactive factors and cells to be studied in future investigations.

## Figures and Tables

**Table 1:** Reaction conditions for oxidation of native alginate with the corresponding degrees of oxidation and substitution.

Amount of NaIO_4_ Used (eq)	Reaction Time (h)	DO_exp_ (%) Titration	DS_CHO_ Titration	DO_exp_ (%) UV/VIS Spectro-Scopy	DS_CHO_ UV/VIS Spectro-Scopy
0.25	3	12.5	0.25	9	0.18
0.25	24	13	0.26	9	0.18
0.5	3	15.5	0.31	12.5	0.25
1	3	21.5	0.43	18	0.36
1.2	24	23.5	0.48	21	0.42
1.2	72	24.5	0.49	22.5	0.45

DO_exp_: experimental degree of oxidation; DS_CHO_: degree of substitution of aldehyde groups; eq: equivalent in relation to the molar amount of the corresponding polysaccharide.

**Table 2:** Reaction conditions for oxidation of native hyaluronic acid with the corresponding degrees of oxidation and substitution.

Amount of NaIO_4_ Used (eq)	Reaction Time (h)	DO_exp_ (%) Titration	DS_CHO_ Titration	DO_exp_ (%) UV/VIS Spectro-Scopy	DS_CHO_ UV/VIS Spectro-Scopy
0.5	6	1	0.02	1	0.02
0.5	24	6	0.12	3.5	0.07
1	6	4	0.08	2.5	0.05
1	24	25.5	0.51	22	0.44
1.2	24	25	0.5	21	0.42
1.2	72	36	0.72	36	0.72

DO_exp_: experimental degree of oxidation; DS_CHO_: degree of substitution of aldehyde groups; eq: equivalent in relation to the molar amount of the corresponding polysaccharide.

**Table 3:** Storage (G’) moduli, loss (G”) moduli, and gelation times of the hydrogels.

Hydrogel	G’ (Pa)	G” (Pa)	Gelation Time (min)
CMC-oALG_DS 0.25_	7 ± 4	5.2 ± 0.9	>30 min
CMC-oALG_DS 0.49_	111 ± 29	8.4 ± 2.2	16 ± 4
CMC-oHA_DS 0.02_	11 ± 9	5.1 ± 2.7	>30 min
CMC-oHA_DS 0.72_	323 ± 16	11.7 ± 1.6	8 ± 4

CMC: carboxymethyl chitosan; G’: shear storage modulus; G”: loss modulus; oALG: oxidized alginate, oHA: oxidized hyaluronic acid. Storage (G’) and loss (G”) moduli were obtained from the frequency sweep measurements at 1 Hz and 0.1% strain (Figure 6C). Gelation time was calculated from the time evolution of the storage modulus in the crosslinking process (Figure 6A). Each value represents an average of three replicates.

## Supplementary Materials

The following are available online at https://www.mdpi.com/2218-273X/10/8/1185/s1, Figure S1: The integral titration curve of amino groups in CMC; Figure S2: Correlation studies between the CHO content of oALG and the metabolic activity of 3T3- L1 fibroblasts; Figure S3: Correlation studies between the CHO content of oHA and the metabolic activity of 3T3-L1 fibroblasts; Table S1: Effect of Polysaccharides Oxidation on Weight Average Molecular Weight (Mw) and Polydispersity Index (PDI) of Alginate (ALG); Table S2: Effect of Polysaccharides Oxidation on Weight Average Molecular Weight (Mw) and Polydispersity Index (PDI) of Hyaluronic acid (HA).

## Abbreviations

ALG	alginate
CFDA	5(6)-carboxyfluoresceine diacetate
CMC	carboxymethyl chitosan
DMEM	Dulbecco’s modified Eagle’s medium
DO_exp_	experimental degree of oxidation
DS_CHO_	degree of substitution of aldehyde groups
EDTA	ethylenediamine tetraacetic acid
eq	equivalent
FBS	fetal bovine serum
FTIR	Fourier-transform infrared spectroscopy
G*	complex modulus
G’	shear storage modulus
G”	loss modulus
GPC	gel permeation chromatography
HA	hyaluronic acid
HPLC	high performance liquid chromatography
ITS	insulin-transferrin-selenium A
Mw	weight average molecular weight
nALG	native alginate
nHA	native hyaluronic acid
oPs	oxidized polysaccharides
PBS	phosphate buffered saline
PEG	polyethylene glycol
Ps	polysaccharides
PSF	penicillin-streptomycin-fungizone
RT	room temperature
SD	standard deviations
UV/VIS spectroscopy	ultraviolet/visible spectroscopy
