# Supplementary material for: Synthesis and Characterization of Oxidized Polysaccharides for In Situ Forming Hydrogels
Source: Biomolecules. 2020 Aug 14;10(8):1185. doi: 10.3390/biom10081185 (PMC7464976; doi:10.3390/biom10081185)
Supplement: Supplementary file 1 [file biomolecules-10-01185-s001.pdf]

Supporting Information

Synthesis and Characterization of Oxidized Polysaccharides for *in Situ* Forming Hydrogels

Muhammad Muhammad [#], Christian Willems [#], Julio Rodríguez-Fernández [†], Gloria Gallego-Ferrer [†,‡] and Thomas Groth[,#,+]*

AUTHOR ADDRESS

[#] Department Biomedical Materials, Martin Luther University Halle-Wittenberg, Heinrich-Damerow-Strasse 4, D-06120 Halle (Saale), Germany, [†] Centre for Biomaterials and Tissue Engineering, Universitat Politècnica de València, Camino de Vera s/n, 46022 Valencia, Spain, [‡] Biomedical Research Networking Centre in Bioengineering, Biomaterials and Nanomedicine (CIBER-BBN), Valencia, Spain, [+] Interdisciplinary Center of Applied Research, Martin Luther University Halle-Wittenberg, D-06099 Halle (Saale), Germany

Corresponding Author

*Prof. Dr. T. Groth

Department Biomedical Materials, Martin Luther University Halle-Wittenberg, Heinrich-Damerow-Strasse 4, D-06120 Halle (Saale), Germany

Interdisciplinary Center of Applied Research, Martin Luther University Halle-Wittenberg, D-06099 Halle (Saale), Germany

Email: thomas.groth@pharmazie.uni-halle.de

Phone: +49-(0)-345-55 28 461

Fax: +49-(0)-345-55 27 379

Number of pages: 7

Number of figures: 3

Number of tables: 3

Table of contents **Page**

1. Quantification of Amino Groups in Carboxymethyl Chitosan (CMC) S3-4

2. Quantitative Results of Gel Permeation Chromatography of Ps and oP S5

3. Quantitative Results of Aldehyde Content of oPs Obtained via Titration S6

4. Correlation Studies between 3T3-L1 Fibroblasts Metabolic Activity and S6-8

Aldehyde Content of oPs

5. References S8

EXPERIMENTAL SECTION

Quantification of Amino Groups in Carboxymethyl Chitosan (CMC)

Amino group content of CMC was determined by using potentiometric titration.[1] In brief, 0.1 g of CMC was dissolved in 25 mL of 0.1 M HCl solution. The ionic strength of the solution was then adjusted to 0.1 M using 0.1 M KCl. Finally, the CMC solution was titrated with 0.1 NaOH. The pH value of the solution was simultaneously recorded under continuous stirring. The obtained data were used to draw the integral curve between the differential of the pH values and the corresponding NaOH volumes.

RESULTS

Quantification of Amino Groups in CMC

HCl reacts with amino groups (NH_2) of CMC to form a quaternary ammonium salt ($R\text{-}NH3^+$) (Cl^-). NaOH was then added to neutralize the solution. By plotting the data obtained from the potentiometric titration of amino groups in CMC, an integral curve with two peaks was obtained.

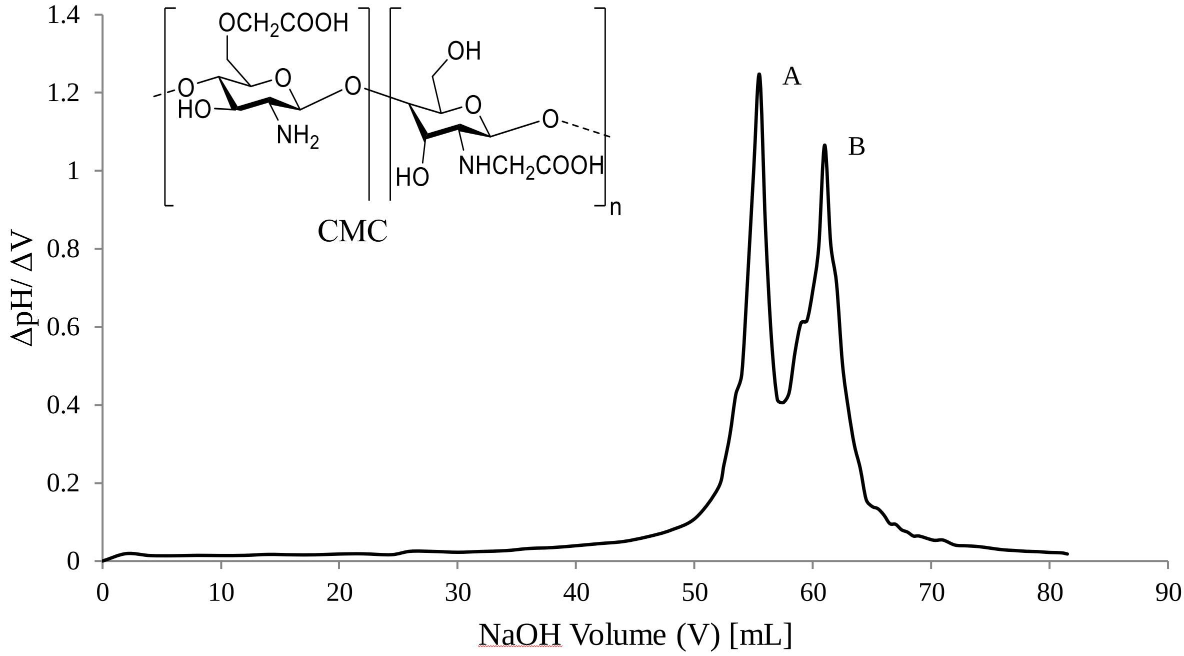

Figure S1. The integral titration curve of amino groups in CMC.

The differential volume of NaOH, between the first A) and second B) neutralization points (V_{neut}), that corresponds to the acid consumed by amino groups present in CMC was calculated (5.5 mL) and used to determine the molar amount of amino groups in CMC, which resulted in 55 $x10^{-4}$ mol g^{-1}. It is crucial to have enough amino groups in CMC in order to form imine bonds with the aldehyde functionality of oPs. This is required for hydrogel formation driven by Schiff base crosslinking reaction.[2]

Quantitative Results of Gel Permeation Chromatography of Ps and oPs

Table S1. Effect of Polysaccharides Oxidation on Weight Average Molecular Weight (Mw) and Polydispersity Index (PDI) of Alginate (ALG).

Sample	Mw (kDa)		PDI	
	**Mean**	**SD**	**Mean**	**SD**
nALG	236	42	2.9	0.2
oALG $_{DS\ 0.25}$	70	5	2.2	0.2
oALG $_{DS\ 0.31}$	46	1	2.1	0.1
oALG $_{DS\ 0.43}$	35	1	1.9	0.1
oALG $_{DS\ 0.49}$	14	5	1.5	0.5

Molecular weights (Mw) and polydispersity indexes (PDI) were determined by gel permeation chromatography (GPC).
The degrees of substitution of aldehyde groups (DS) were obtained from titration; SD: standard deviations.

Table S2. Effect of Polysaccharides Oxidation on Weight Average Molecular Weight (Mw) and Polydispersity Index (PDI) of Hyaluronic acid (HA).

Sample	Mw (kDa)		PDI	
	**Mean**	**SD**	**Mean**	**SD**
nHA	1224	25	2.1	0.9
oHA $_{DS\ 0.02}$	148	3	2.5	0.1
oHA $_{DS\ 0.12}$	55	1	2.2	0.1
oHA $_{DS\ 0.08}$	66	2	2.3	0.1
oHA $_{DS\ 0.51}$	30	4	1.7	0.1
oHA $_{DS\ 0.72}$	17	1	1.7	0.1

Molecular weights (Mw) and polydispersity indexes (PDI) were determined by gel permeation chromatography (GPC).
The degrees of substitution of aldehyde groups (DS) were obtained from titration; SD: standard deviations.

Quantitative Results of Aldehyde Content of oPs Obtained via Titration

Table S3. Aldehyde Contents of Oxidized Polysaccharides.

oPs	CHO content ($x10^{-4}$ mol g^{-1}) Titration
oALG $_{DS\ 0.25}$	14.2
oALG $_{DS\ 0.31}$	18.1
oALG $_{DS\ 0.43}$	25.2
oALG $_{DS\ 0.49}$	28.2
oHA $_{DS\ 0.02}$	0.53
oHA $_{DS\ 0.08}$	2.1
oHA $_{DS\ 0.51}$	13.2
oHA $_{DS\ 0.72}$	18.8

The degrees of substitution of aldehyde groups (DS) were obtained from titration.

Correlation Studies between 3T3-L1 Fibroblasts' Metabolic Activity and Aldehyde Content of oPs

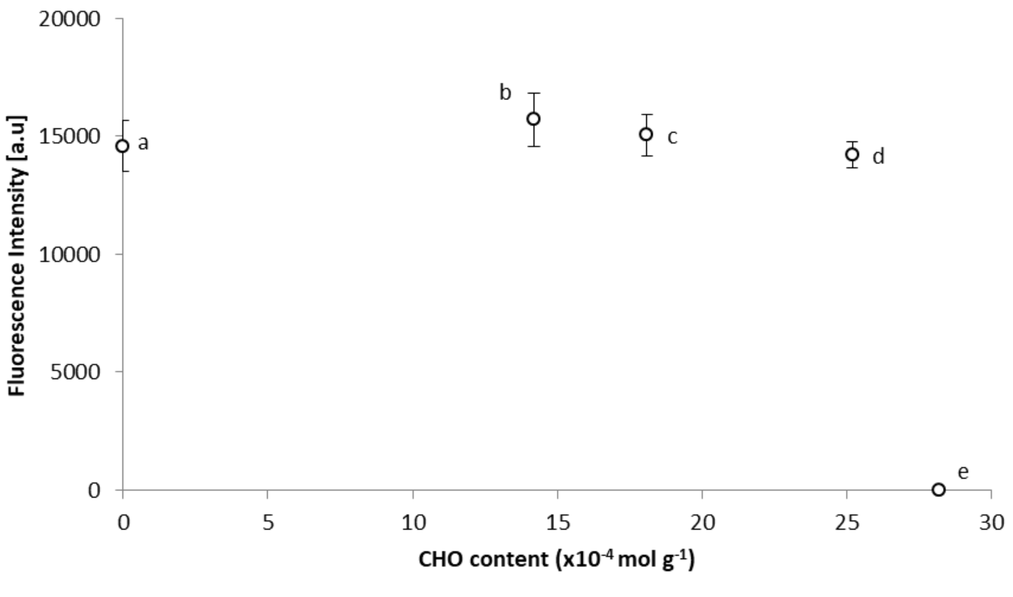

Figure S2. Correlation studies between the CHO content of oALG and the metabolic activity of 3T3- L1 fibroblasts.

a: nALG; b: oALG $_{DS\ 0.25}$; c: oALG $_{DS\ 0.31}$; d: oALG $_{DS\ 0.43}$; e: oALG $_{DS\ 0.49}$.

Error bars in the X axis represent the standard deviations of the CHO contents of oALG. (Error bars are not visible in the figure above, because their corresponding values were small).

Error bars in the Y axis represent the standard deviations of the fluorescence intensities of 3T3-L1 fibroblasts' metabolic activity.

As the figure depicts, there is an insignificant negative relationship between the CHO content of oALG and the metabolic activity of 3T3-L1 fibroblasts obtained from Qblue assay, r = -0.57, p = 0.32 > 0.05.

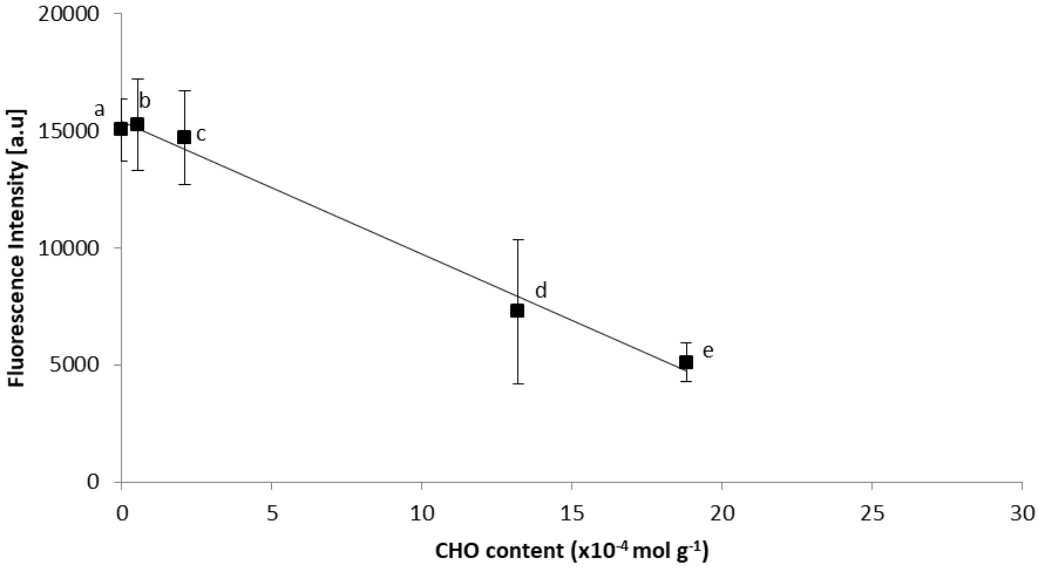

Figure S3. Correlation studies between the CHO content of oHA and the metabolic activity of 3T3-L1 fibroblasts.

a: nHA; b: oHA $_{DS\ 0.02}$; c: oHA $_{DS\ 0.08}$; d: oHA $_{DS\ 0.51}$; e: oHA $_{DS\ 0.72}$.

Error bars in the X axis represent the standard deviations of the CHO contents of oHA. (Error bars are not visible in the figure above, because their corresponding values were small).

Error bars in the Y axis represent the standard deviations of the fluorescence intensities of 3T3-L1 fibroblasts' metabolic activity.

As the figure shows, there is a significant negative relationship between the CHO content of oHA and the metabolic activity of 3T3-L1 fibroblasts obtained from Qblue assay, $r = -0.99$, $p = 0.0004 < 0.05$.

REFERENCES

1. Hussain, R.; Iman, M.; Maji, T. K. Determination of Degree of Deacetylation of Chitosan and Their Effect on the Release Behavior of Essential Oil from Chitosan and Chitosan- Gelatin Complex Microcapsules. **2013**, 10.
2. Radhakrishnan, J.; Subramanian, A.; Krishnan, U. M.; Sethuraman, S. Injectable and 3D Bioprinted Polysaccharide Hydrogels: From Cartilage to Osteochondral Tissue Engineering. *Biomacromolecules* **2017**, *18* (1), 1–26. https://doi.org/10.1021/acs.biomac.6b01619.
